# Outcome of adjuvant radiotherapy after total hysterectomy in patients with uterine leiomyosarcoma or carcinosarcoma: a SEER-based study

**DOI:** 10.1186/s12885-019-5879-7

**Published:** 2019-07-15

**Authors:** Yuan Li, Haitao Ren, Junmei Wang

**Affiliations:** 1 Department of Ultrasound, Women’s Hospital, School of Medicine, Zhejiang University, Hangzhou, 310006 China; 2 Department of Burns and Wound Care Center, the Second Affiliated Hospital, School of Medicine, Zhejiang University, Hangzhou, 310009 China

**Keywords:** Adjuvant radiotherapy, Carcinosarcoma, Leiomyosarcoma, SEER, Uterine sarcoma

## Abstract

**Background:**

The clinical impact of adjuvant radiotherapy on uterine sarcoma is unclear, and may depend on the histological type. Hence, the aim of this study was to evaluate clinical outcomes of adjuvant radiotherapy after total hysterectomy in patients with leiomyosarcoma or carcinosarcoma.

**Methods:**

Data were obtained from the Surveillance, Epidemiology, and End Results (SEER) program. Cox proportional hazards regression analyses were performed to identify risk factors for overall mortality and cancer-specific mortality. In addition, a 1:1 propensity score matching approach was performed, in which age group, disease stage, tumor grade, tumor size, and lymphadenectomy status were matched.

**Results:**

A total of 566 leiomyosarcoma and 1069 carcinosarcoma patients with stage I-III disease were included. Both regular Cox regression analysis and propensity score matching analysis revealed that utilization of adjuvant radiotherapy did not affect overall and cancer-specific mortality in patients with leiomyosarcoma. In contrast, for patients with carcinosarcoma, total mortality risk was significantly decreased with EBRT, brachytherapy, and combination radiotherapy compared with no radiotherapy. Cancer-specific mortality risk was significantly decreased with brachytherapy and combination radiotherapy as compared with no radiotherapy. Propensity score matching analyses revealed similar results in overall mortality, but not cancer-specific mortality, in patients with carcinosarcoma. Furthermore, the frequency of patients who did not receive any form of adjuvant radiotherapy was four times higher than those underwent adjuvant radiotherapy.

**Conclusions:**

Adjuvant radiotherapy may provide a survival benefit for uterine carcinosarcoma, but not leiomyosarcoma. In addition, adjuvant radiotherapy is underutilized, and increased utilization of adjuvant radiotherapy may improve the survival rate of patients with carcinosarcoma.

**Electronic supplementary material:**

The online version of this article (10.1186/s12885-019-5879-7) contains supplementary material, which is available to authorized users.

## Background

Uterine sarcomas are rare malignant tumors arose from the smooth muscle or connective tissue of the uterus, which account for only 3 to 7% of uterine malignancies [1] but are the most common cause of uterine cancer-specific death [2]. Uterine sarcomas are histologically heterogeneous; two major histological subtypes are uterine leiomyosarcomas and carcinosarcomas that account for 30 and 50% of uterine sarcomas, respectively [1]. Leiomyosarcomas are smooth muscle tumors, expressing desmin, h-caldesmon, and smooth muscle α actin [1]. In contrast, carcinosarcomas are biphasic neoplasms consisting of admixed epithelial and mesenchymal elements, but are mostly of monoclonal origin [1, 3]. According to conversion theory, such biphasic tumors are likely as a result of late divergence and metaplasia of the carcinomatous component into the sarcomatous components [3, 4]. Leiomyosarcomas often occurred in women aged 40 or older, and carcinosarcomas occurred in women who were much older [1].

Both uterine leiomyosarcomas and carcinosarcomas are highly aggressive with poor prognosis. Because of hematogenous spread, uterine leiomyosarcomas metastasize early primarily to the lungs [5]. In addition, at the time of diagnosis, one third of carcinosarcoma cases have already spread beyond the uterus [3]. Recurrence rate of leiomyosarcomas ranges from 53 to 71% [1], while recurrence rate of carcinosarcomas is 64% [3]. The 5-year overall survival (OS) rate was 15 to 20 and 30% for leiomyosarcomas and carcinosarcoma, respectively [6–8].

The primary curative treatment for uterine sarcomas is total abdominal hysterectomy, while debulking is used for palliative purpose if there is tumor outside of the uterus [5, 6]. Removal of the ovaries and lymph node may be performed if disease is present; however, removal in all cases is debatable [6–8]. Survival in the presence of lymph node metastasis is significantly lower than if there is no metastasis to the lymph nodes; however, it is unclear if lymphadenectomy offers survival benefits [7–10].

The effect of adjuvant radiotherapy on uterine sarcoma is unclear, and may depend on the histological type [6, 11, 12]. Several retrospective studies have suggested that postoperative radiation decreases the local recurrence rate, but does not improve OS in patients with uterine sarcoma [6, 11–13]. In contrast, some studies have indicated that adjuvant radiotherapy does provide survival benefits for patients with leiomyosarcoma or carcinosarcoma [9, 11, 12, 14]. Furthermore, using competing risk modeling, Wong et al. (2013) demonstrated that adjuvant radiotherapy may improve both local recurrence rate and OS in patients with leiomyosarcoma [15]. The phase III EORTC trial that evaluated efficacy of adjuvant radiotherapy in patients with stage I-II uterine sarcomas found that adjuvant radiotherapy reduces any local recurrence rate by 37.5 and 48.9% in patients with leiomyosarcoma and carcinosarcoma, respectively [16].

To examine the hypothesis that the histological type of uterine sarcomas influences the effect of adjuvant postoperative radiotherapy on mortality in patients with uterine sarcoma, a population-based study was conducted, focusing on two major histological types, uterine leiomyosarcomas and carcinosarcomas. Thus, the present study aimed to evaluate the effect of adjuvant radiotherapy after total hysterectomy on overall mortality and cancer-specific mortality in patients with leiomyosarcoma or carcinosarcoma using data from the US National Cancer Institute (NCI) Surveillance, Epidemiology, and End Results (SEER) program database.

## Methods

### Data source

Data for this study were extracted from the US NCI SEER database that was released in April 2016, based on the November 2015 submission [17]. The SEER Data are collected from population-based registries, which cover approximately 28% of the US population. The SEER database contains information on patients’ demographics, cancer incidence, primary tumor site, tumor morphology, stage at diagnosis, treatment, and follow-up status. The database is also linked to information on Medicare enrolment and Medicare claims, along with healthcare utilization and cost information for beneficiaries with cancer in the US.

Because all SEER data are de-identified, this study does not require Institutional Review Board approval, or informed consent by the study subjects. While, we obtained permission to access the SEER program data from the US National Cancer Institute (reference number: 11770-Nov2016).

### Study population

The SEER database was examined to identify patients with primary uterine sarcoma according to codes of the International Classification of Diseases for Oncology (ICD-O) for anatomic sites (PRIMSITE = C54.0-C54.3, C54.8-C54.9, C55.9) who underwent total hysterectomy (SURGPRIF = 40, 50). Of them, patients with stage IV or unknown stage were excluded. Of the uterine sarcoma patients identified, patients with two major histological subtypes, leiomyosarcoma (HISTO3V = 8890, 8891, 8896) and carcinosarcoma (HISTO3V = 8980, 8981), were selected for the final analysis, because these 2 histological subtypes account for more than 75% of uterine sarcoma cases [1, 6]. The SEER coding system classifies leiomyosarcoma into 4 grades: grade 1, well differentiated; grade 2, moderately differentiated; grade 3, poorly differentiated; and undifferentiated/anaplastic.

### Data extraction

Data obtained from the SEER database included patients’ age at diagnosis, race, year of diagnosis, American Joint Committee on Cancer (AJCC) stage and grade, tumor size, treatments, and survival status (alive, death due to sarcoma, death due to other causes). Regarding adjuvant postoperative radiotherapy, patients were classified into four groups: i) no postoperative radiotherapy (RAD_SURG = 0 or RADIATN =0); ii) external beam radiation therapy (EBRT) alone (RAD_SURG = 3 and RADIATN =1); iii) brachytherapy (radioactive implants or radioisotopes) alone (RAD_SURG = 3 and RADIATN =2 or 3); iv) combination radiotherapy (combination of EBRT and either radioactive implants or radioisotopes; RAD_SURG = 3 and RADIATN = 4). Patients were also categorized according to lymphadenectomy status: yes, no, or unknown. Coding details and rules followed guidelines established by the SEER program (https://seer.cancer.gov).

### Statistical analysis

Categorical data were presented as count and percentage. Univariate and multivariate Cox proportional hazards regressions were performed to identify potential risk factors for overall mortality and cancer-specific mortality in patients with leiomyosarcoma or carcinosarcoma. Significant variables found in the univariate analysis (*P* < 0.05) were input into the multivariate Cox proportional hazards regression analysis using the stepwise selection method. To better balance patients who did and did not get radiation, a 1:1 propensity score matching approach was performed for an additional analysis, in which age group, disease stage, tumor grade, tumor size, and lymphadenectomy status were matched.

For overall mortality analysis, any cause of death was treated as an event, and survivors were treated as censors. For cancer-specific mortality analysis, deaths due to uterine sarcoma were events, and deaths from other causes or survivors were censors. Data were presented as adjusted hazard ratio (aHR) and 95% confidence interval (CI) and *P*-value. To identify trends of adjuvant radiotherapy usage, the frequencies of adjuvant radiotherapy usage during the study period were counted. The significant level was set to 0.05. Statistical analyses were performed by SAS statistical software (version 9.4, SAS Inc., Cary, NC, USA) and R software (version 3.2.2).

## Results

### Study population

A total of 4331 patients, who were diagnosed with uterine leiomyosarcoma or carcinosarcoma and underwent total hysterectomy, were identified in the SEER database during the period from 2004 to 2013. After excluding patients with AJCC stage IV or missing stage, 1635 patients were included in the final analysis.

### Patients’ characteristics

Patients’ characteristics are summarized in Table 1. Of the 1635 included patients, 1028 (62.9%) were over 60 years of age. Most of the patients were white (71.1%), had stage I disease (65.4%), and had a tumor size > 50 mm (59.6%). The most frequently used adjuvant radiotherapy was EBRT that was applied to 14.8% of patients, followed by brachytherapy (9.7%) and combination radiotherapy (5.8%). However, 68.3% of the total population did not receive any form of adjuvant radiotherapy (Table 1).

**Table 1:** Patients’ characteristics

	All	Leiomyosarcoma	Carcinosarcoma
	*N* = 1635	*n* = 566	*n* = 1069
Age ≥ 60 years	1028 (62.9)	175 (30.9)	1934 (79.8)
Race			
White	1162 (71.1)	397 (70.1)	765 (71.6)
Black	324 (19.8)	111 (19.4)	214 (20.0)
Other ^a^	145 (8.9)	56 (9.9)	89 (8.3)
Unknown	4 (0.2)	3 (0.5)	1 (0.1)
Year of diagnosis after 2009	1635 (100)	566 (100.0)	1069 (100.0)
AJCC Stage			
Stage I	1070 (65.4)	443 (78.3)	627 (58.7)
Stage II	153 (9.4)	66 (11.7)	87 (8.1)
Stage III	412 (25.2)	57 (10.0)	355 (33.2)
Grade			
Grade 1	34 (2.1)	21 (3.7)	13 (1.2)
Grade 2	81 (5.0)	53 (9.3)	28 (2.6)
Grade 3	539 (33.0)	91 (16.1)	448 (41.9)
Undifferentiated/anaplastic	380 (23.2)	146 (25.8)	234 (21.9)
Unknown	601 (36.7)	255 (45.1)	346 (32.4)
Tumor size			
≤ 50 mm	461 (28.2)	87 (15.4)	374 (35.0)
> 50 mm	974 (59.6)	424 (74.9)	550 (51.5)
Unknown	200 (12.2)	55 (9.7)	145 (13.5)
Lymphadenectomy			
No	583 (35.7)	374 (66.1)	209 (19.6)
Yes	1025 (62.7)	185 (32.7)	840 (78.6)
Unknown	27 (1.7)	7 (1.2)	20 (1.8)
Adjuvant radiation			
No treatment	1117 (68.3)	476 (84.1)	641 (60.0)
EBRT alone	242 (14.8)	64 (11.3)	178 (16.7)
Brachytherapy alone	159 (9.7)	11 (1.9)	148 (13.8)
Combination radiotherapy ^b^	95 (5.8)	13 (2.3)	82 (7.7)
Others	22 (1.4)	2 (0.4)	20 (1.8)

*AJCC* American Joint Committee on Cancer, *EBRT* external beam radiation therapy

Data are presented as number (percentage)

^a^Including American Indian, Alaska native, Asian Pacific Islander, and other unspecified

^b^Combination radiotherapy is EBRT with radioactive implants

Among 1635 eligible patients, 566 patients had leiomyosarcoma and 1069 had carcinosarcoma (Table 1). A large proportion of patients with leiomyosarcoma had undifferentiated/anaplastic tumors (25.8%), and did not undergo a lymphadenectomy (66.1%). In contrast, most patients with carcinosarcoma had grade 3 tumors (41.9%), and 78.6% of patients underwent lymphadenectomy (Table 2).

**Table 2:** Multivariate Cox proportional hazards regression analyses for overall and cancer-specific mortality in patients with leiomyosarcoma or carcinosarcoma

	Leiomyosarcoma				Carcinosarcoma			
	Overall ^a^		Cancer-specific ^b^		Overall ^c^		Cancer-specific ^d^	
Variables	aHR (95% CI)	*P*-value	aHR (95% CI)	*P*-value	aHR (95% CI)	*P*-value	aHR (95% CI)	*P*-value
Adjuvant radiotherapy								
No treatment	reference		reference		reference		reference	
EBRT alone	0.90 (0.54, 1.51)	0.697	0.91 (0.51, 1.64)	0.760	**0.72 (0.53, 0.99)**	**0.042**	0.78 (0.54, 1.14)	0.199
Brachytherapy alone	0.57 (0.14, 2.31)	0.429	0.47 (0.07, 3.38)	0.452	**0.55 (0.37, 0.80)**	**0.002**	**0.51 (0.31, 0.84)**	**0.009**
Combination radiotherapy ^e^	NA	NA	NA	NA	**0.47 (0.29, 0.77)**	**0.003**	**0.53 (0.29, 0.95)**	**0.034**
Age								
< 60 years	reference				reference			
≥ 60 years	**1.55 (1.11, 2.18)**	**0.011**			**1.74 (1.27, 2.37)**	**0.001**		
AJCC Stage								
Stage I	reference		reference		reference		reference	
Stage II	**1.82 (1.15, 2.89)**	**0.011**	**1.85 (1.09, 3.12)**	**0.022**	1.06 (0.67, 1.69)	0.802	1.10 (0.61, 1.98)	0.758
Stage III	**3.07 (2.01, 4.69)**	**< 0.001**	**3.21 (2.02, 5.13)**	**< 0.001**	**2.36 (1.88, 2.97)**	**< 0.001**	**2.57 (1.93, 3.42)**	**< 0.001**
Grade								
Grade 1	reference							
Grade 2	1.58 (0.18, 13.60)	0.679						
Grade 3	7.17 (0.97, 52.86)	0.053						
Undifferentiated/anaplastic	5.08 (0.69, 37.09)	0.110						
Tumor size								
≤ 50 mm			reference		reference		reference	
> 50 mm			**2.98 (1.38, 6.45)**	**0.006**	**1.96 (1.50, 2.57)**	**< 0.001**	**2.07 (1.46, 2.93)**	**< 0.001**
Lymphadenectomy								
No					reference		reference	
Yes					**0.61 (0.47, 0.78)**	**< 0.001**	**0.61 (0.45, 0.84)**	**0.003**

*aHR* adjusted hazard ratio, *CI* confidence interval, *EBRT* external beam radiation therapy, *AJCC* American Joint Committee on Cancer

^a^Model was adjusted by age group, and stage, grade

^b^Model was adjusted by stage, and tumor size

^c^Model was adjusted by age group, stage, tumor size, and lymphadenectomy

^d^Model was adjusted stage, tumor size, and lymphadenectomy

^e^Combination radiotherapy: combination of EBRT and either radioactive implants or radioisotopes

NA, not available because no patient died during the study period

Unknown data for adjuvant radiotherapy, AJCC stage, grade, tumor size, and lymphadenectomy status were not shown in the table

Bold text indicates a significant difference with a *p*-value less than 0.05 compared to the reference group

**Fig. 1:**
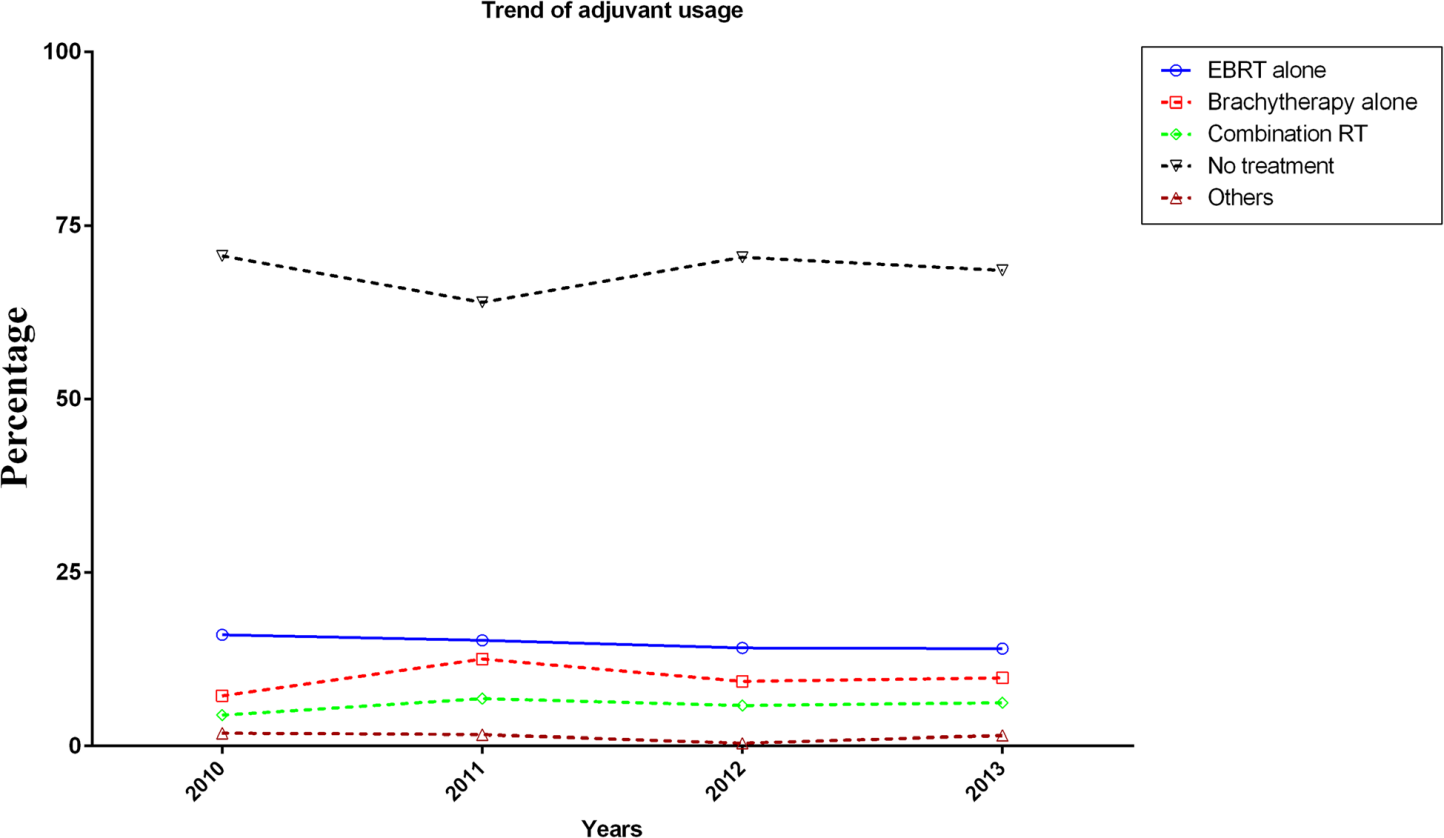
Trend of adjuvant radiation usage. Utilization of adjuvant radiation per year was presented in percentage

The trend of adjuvant radiotherapy usage during the study period is shown in Fig. 1. Since patients with stage IV or unknown stage were excluded, the number of eligible patients in 2004–2009 was zero. As a result, only the period of 2010–2013 was shown in Fig. 1. During 2010–2013, the frequency of patients who did not receive any form of adjuvant radiotherapy was four times higher than those underwent adjuvant radiotherapy (Fig. 1).

During the study period, a total of 478 patients died. Of these 325 deaths were caused by uterine sarcoma, while others were not specified in the SEER database. The median survival time for the total population was 15 months (interquartile range [IQR]: 7–27 months). During the study period, 141 patients with leiomyosarcoma died, and only 111 out of 141 deaths were caused by leiomyosarcoma. The median survival time of all leiomyosarcoma patients was 16.5 months (IQR: 8–28 months). In contrast, 337 patients with carcinosarcoma died, and only 214 out of 337 deaths were caused by carcinosarcoma. The median survival time of all carcinosarcoma patients was 15 months (IQR: 6–26 months).

### Cox proportional hazards regression for overall and cancer-specific mortality

Cox proportional hazards regression analyses were then performed to identify risk factors for overall and cancer-specific mortality in patients with leiomyosarcoma or carcinosarcoma. The results of the univariate Cox proportional hazards regression analyses of the associations of overall and cancer-specific mortality with adjuvant radiotherapy, demographic and clinical factors in patients with leiomyosarcoma or carcinosarcoma are shown in Additional file 1: Table S1.

Multivariate analyses revealed that for patients with leiomyosarcoma, there was no significant difference in overall and cancer-specific mortality with the use of any type of adjuvant radiotherapy as compared with no radiotherapy (Table 2). In contrast, for patients with carcinosarcoma, the risk of overall mortality was significantly decreased with EBRT alone (aHR = 0.72, 95% CI: 0.53, 0.99, *P* = 0.042), brachytherapy alone (aHR = 0.55, 95% CI: 0.37, 0.80, *P* = 0.002), and combination radiotherapy (aHR = 0.47, 95% CI: 0.29, 0.77, *P* = 0.003) as compared with no radiotherapy (Table 2). In addition, for patients with carcinosarcoma, the risk of cancer-specific mortality was significantly decreased with brachytherapy alone (aHR = 0.51, 95% CI: 0.31, 0.84, *P* = 0.009), combination radiotherapy (aHR = 0.53, 95% CI: 0.29, 0.95, *P* = 0.034) as compared to no radiotherapy (Table 2).

In addition, for patients with leiomyosarcoma, older age and higher stage were significantly associated with elevated risk of overall mortality, while higher stage (stage II and III) and larger tumor size were significantly associated with increased risk of cancer-specific mortality (all *p* ≤ 0.022, Table 2). For patients with carcinosarcoma, higher age, stage III, and larger tumor size increased risk for overall mortality, but lymphadenectomy decreased risk of overall mortality (all *p* ≤ 0.001, Table 2). Furthermore, stage III and larger tumor size elevated risk of cancer-specific mortality, whereas lymphadenectomy lower risk of cancer-specific mortality (all *p* ≤ 0.003, Table 2).

### Overall and cancer-specific mortality in 1:1 propensity score matching sample

To better balance the patients who did and did not receive radiotherapy, the associations between mortality and adjuvant radiotherapy type were also assessed by Cox regression model in the 1:1 propensity score matching sample. Propensity score matching results indicated that for patients with leiomyosarcoma there was no significant difference in overall and cancer-specific mortality with the use of any type of adjuvant radiotherapy as compared with no radiotherapy (Table 3). For patients with carcinosarcoma, propensity score matching showed the risk of overall mortality was significantly decreased with EBRT alone (aHR = 0.65, 95% CI: 0.45, 0.93, *P* = 0.020), brachytherapy alone (aHR = 0.62, 95% CI: 0.40, 0.95, *P* = 0.029), and combination radiotherapy (aHR = 0.47, 95% CI: 0.26, 0.85, *P* = 0.013) as compared with no radiotherapy (Table 3). However, no significant difference in cancer-specific mortality with the use of any type of adjuvant radiotherapy as compared with no radiotherapy in patients with carcinosarcoma was observed (Table 3).

**Table 3:** Cox proportional hazards regression for overall and cancer-specific mortality of leiomyosarcoma and carcinosarcoma in the 1:1 propensity score matching sample

	Leiomyosarcoma (*n* = 180)				Carcinosarcoma (*n* = 856)			
	Overall ^b^		Cancer-specific ^c^		Overall ^b^		Cancer-specific ^c^	
Variables	HR (95% CI)	*P*-value	aHR (95% CI)	*P*-value	HR (95% CI)	*P*-value	aHR (95% CI)	*P*-value
Adjuvant radiotherapy								
No treatment	reference		reference		reference		reference	
EBRT alone	0.71 (0.33, 1.54)	0.386	0.69 (0.27, 1.79)	0.447	**0.65 (0.45, 0.93)**	**0.020**	0.78 (0.50, 1.22)	0.274
Brachytherapy alone	0.68 (0.14, 3.30)	0.633	0.45 (0.05, 3.93)	0.469	**0.62 (0.40, 0.95)**	**0.029**	0.60 (0.34, 1.05)	0.074
Combination radiotherapy ^a^	NA	NA	NA	NA	**0.47 (0.26, 0.85)**	**0.013**	0.67 (0.33, 1.33)	0.250

*aHR* adjusted hazard ratio, *CI* confidence interval, *EBRT* external beam radiation therapy

^a^Combination radiotherapy: combination of EBRT and either radioactive implants or radioisotopes

^b^Univariate regression model

^c^Race was adjusted for in multivariate regression model

NA: not available because no patient died during the study period

Unknown data for adjuvant radiotherapy were not shown in the table

Bold text indicates a significant difference with a *p*-value less than 0.05 compared to the reference group

Furthermore, non cancer-specific survival was analyzed and the results were presented in Additional file 1: Tables S2 and S3 (comparison between patients with and without adjuvant radiotherapy). For patients with leiomyosarcoma, their non cancer-specific survivals were not significantly different between those with or without adjuvant radiotherapy (Additional file 1: Table S3). However, the significant difference in non cancer-specific survival between those with or without adjuvant radiotherapy was observed in patients with carcinosarcoma (Additional file 1: Tables S2 and S3).

## Discussion

The present study used the US NCI SEER database to determine the effect of adjuvant radiotherapy on survival of patients with uterine leiomyosarcoma or carcinosarcoma. The results of the standard Cox proportional regression analyses showed that there was no difference in overall mortality or cancer-specific mortality in patients with leiomyosarcoma whether or not any type of radiotherapy was used. Whereas in patients with carcinosarcoma overall mortality risk was significantly decreased with EBRT alone, brachytherapy alone, and combination radiotherapy as compared to no radiotherapy, and the risk of cancer-specific mortality was significantly decreased with brachytherapy alone and combination radiotherapy as compared to no radiotherapy. The results of propensity score matching analyses revealed that no association between mortality and the use of adjuvant radiotherapy in patients with leiomyosarcoma. In contrast, EBRT alone, brachytherapy alone, and combination radiotherapy significantly lowered the risk of overall mortality, but not cancer-specific mortality, in patients with carcinosarcoma. The results of the present study suggest that adjuvant radiotherapy might be underutilized, and proper use of adjuvant radiotherapy after surgery might improve survival of patients with uterine carcinosarcoma.

Uterine sarcomas are uncommon, highly aggressive tumors, while definitive guidelines for their management have yet to be determined. This is especially true with respect to adjuvant therapy with studies providing varying results as to its effectiveness [2, 18]. By analyzing SEER database (2002–2012), Hosh et al. reported that among 13,089 cases of uterine sarcoma, carcinosarcoma (53%) was the most commonly diagnosed subtype followed by leiomyosarcoma (24%) and stromal sarcoma (14%), and patients with leiomyosarcoma and stromal sarcoma had significantly lower overall mortality than those with carcinosarcoma (HR = 0.88, 95% CI: 0.83–0.94, and HR = 0.67, 95% CI: 0.67–0.73, respectively) [6]. In addition, the overall mortality was significantly better in patients who had surgery with radiation than those who had surgery alone (HR = 0.89, 95% CI: 0.83, 0.95) [6]. Compared to the study of Hosh et al. [6], the present SEER-based study analyzed SEER database (2004–2013), estimated both overall morality and cancer-specific mortality, and focused on leiomyosarcoma or carcinosarcoma only but not all subtypes of uterine sarcoma. Regarding adjuvant radiotherapy, Hosh et al. [6] focused on the use of EBRT, whereas the present study included EBRT, brachytherapy, and combination radiotherapy. Furthermore, the present study performed propensity score matching analyses, so the corresponding results representing better balance the patients who did and did not receive adjuvant radiotherapy.

Sampath et al. [12] performed a retrospective analysis of 3650 patients with uterine sarcoma who were identified in the Impac Medical Systems (Sunnyvale, CA, USA) National Oncology Database, and found that in the whole cohort adjuvant radiotherapy was not predictive of OS, whereas in patients who did not have metastasis but underwent definitive surgery, adjuvant radiotherapy was associated with longer locoregional failure-free survival. Sampath et al. [12] defined adjuvant radiotherapy as postoperative EBRT with or without brachytherapy. Overall, the authors concluded that the use of adjuvant radiotherapy reduced the risk of locoregional failure at 5 years by 53% [12]. Cha et al. [19] examined the use of radiotherapy in 235 patients with primary uterine carcinosarcoma, of which 41% received adjuvant radiotherapy. The locoregional failure rate was lower for patients who received adjuvant radiotherapy than for those who did not (17.5% vs. 28.5%, *P* = 0.107), and adjuvant radiotherapy was associated with longer locoregional recurrence-free survival in patients who did not undergo pelvic lymph node dissection (52.7% vs. 18.7%, *P* < 0.001) [19]. Moreover, a study of 141 stage I-III uterine sarcoma patients demonstrated that postoperative radiotherapy, composing both EBRT and brachytherapy, with a total dose higher than 50 Gy significantly reduced the local recurrence rate [20].

Our results showed no difference in overall mortality or cancer-specific mortality in patients with leiomyosarcoma regardless whether or not any type of radiotherapy was used. In contrast, the regular Cox regression analyses revealed that in patients with carcinosarcoma overall mortality and cancer-specific mortality were decreased with the use of adjuvant radiotherapy, although the significant differences in cancer-specific mortality were no longer present in the propensity score matching analyses. These findings are consistent with recent evidence that leiomyosarcoma and carcinosarcoma have different molecular characteristics, which may be responsible for their different susceptibility to radiation [21, 22].

Nemani et al. [13] analyzed the SEER data of 1855 patients with uterine carcinosarcoma and found that lymphadenectomy was associated with improved OS in patients with stage I-III disease as compared to no lymphadenectomy. However, they also reported that and adjuvant radiotherapy was not associated with any increase in survival [13]. On the contrary, Clayton Smith et al. [14] also used SEER data to analyze 2461 women with carcinosarcoma, of who 890 received adjuvant radiotherapy. For women who received adjuvant radiotherapy as compared to those who did not, the 5-year OS rates were 41.5 and 33.2%, respectively (*P* < 0.001), and uterine-specific survival rates were 56.0 and 50.8%, respectively (*P* = 0.005) [14]. Radiotherapy was associated with better OS in patients with stage I–III disease (HR = 0.87, *P* = 0.03), and with better OS (HR = 0.63, *P* < 0.001) and uterine-specific survival (HR = 0.63, *P* = 0.004) in patients with stage IV disease [14].

There is no adjuvant therapy that has been consistently shown to improve outcomes for patients with leiomyosarcoma, and as such there are no clear guidelines for treatment [23]. A retrospective review of 208 patients with leiomyosarcoma reported that adjuvant radiotherapy did not improve survival outcomes [9]. A study of SEER data that included approximately 3000 patients from 1988 to 2004 with stage I and II disease found that adjuvant radiotherapy had no effect on survival for early-stage leiomyosarcoma, but that adjuvant radiotherapy reduced the risk of death by 21% in women with carcinosarcoma [23]. In patients with carcinosarcoma, adjuvant radiotherapy substantially reduced mortality in women who did not undergo lymph node dissection, but in node-negative women [23]. Consistently, our results also demonstrated that use of adjuvant radiotherapy was associated with reduced overall and cancer-specific mortality in patients with carcinosarcoma.

However, the opposite results have been also reported. Mahdavi et al. [11] reviewed 147 patients with leiomyosarcoma treated at 11 regional medical centers from 1985 to 2005 and found the 5-year survival of patients who received radiotherapy was significantly higher than those who did not (70% vs. 35%); however, the survival advantage was no longer present at 7.5 years. In addition, the pelvic recurrence rate was lower in the radiotherapy group (18% vs. 49%, *P* = 0.02) [11].

This study has inherent strengths and limitations. The SEER data has been found to be generalizable to the US population [24], and as it is a high-quality national database discrepancies and potential biases are reduced. The SEER database, however, does not contain information of comorbidities, lifestyle and risk factors, environmental exposure, and family history, and thus these factors could not be examined. The SEER database also does not include data on quality of surgery (morcellation), margins, radiation dose and target location, cohort of patients treated prior to the Food & Drug Administration (FDA) notice on uterine tumor morcellation, reliability of cancer specific survival, and cause of death reports. Furthermore, the SEER database also suffers several limitations, such as missing data on grade and stage, unreliable information on cause of death, and potential selection bias. Chemotherapy is also used for the adjuvant treatment of uterine sarcomas, and may improve survival [25]. However, the SEER database does not incorporate information on the use of chemotherapy. This could potentially affect the results as we could not include chemotherapy administration in the analysis. Furthermore, we did not examine outcomes with neoadjuvant radiotherapy. Outcome estimates are different when different staging systems are used (AJCC or International Federation of Gynecology and Obstetrics [FIGO]), and this was not examined [26, 27].

## Conclusions

The results of this population-based study demonstrated that the clinical impact of adjuvant radiotherapy is histological type-specific, with significant decreased overall mortality rate in patients with uterine carcinosarcoma, but not uterine leiomyosarcoma. In addition, adjuvant radiotherapy was underutilized, and increased use of adjuvant radiotherapy might improve the survival rate of patients with carcinosarcoma.

## Additional file

Additional file 1: **Table S1.** Univariate Cox proportional hazards regression for overall and cancer-specific mortality of leiomyosarcoma and carcinosarcoma. **Table S2.** Multivariate Cox proportional hazards regression for non cancer-specific mortality of leiomyosarcoma or carcinosarcoma in 1:1 propensity score matching sample. **Table S3.** Multivariate Cox proportional hazards regression for non cancer-specific mortality of leiomyosarcoma or carcinosarcoma in 1:1 propensity score matching sample. (DOCX 22 kb)

## Data Availability

Data for this study were obtained from the US NCI SEER database (https://seer.cancer.gov) that was released in April 2016, based on the November 2015 submission.

## Abbreviations

aHR: Adjusted hazard ratio
AJCC: American Joint Committee on Cancer
CI: Confidence interval
EBRT: External beam radiation therapy
FDA: Food & Drug Administration
FIGO: International Federation of Gynecology and Obstetrics
ICD-O: International Classification of Diseases for Oncology
IQR: Interquartile range
NCI: National Cancer Institute
OS: Overall survival
SEER: Surveillance, Epidemiology, and End Results
