# Supplementary material for: Outcome of adjuvant radiotherapy after total hysterectomy in patients with uterine leiomyosarcoma or carcinosarcoma: a SEER-based study
Source: BMC Cancer. 2019 Jul 15;19:697. doi: 10.1186/s12885-019-5879-7 (PMC6631553; doi:10.1186/s12885-019-5879-7)
Supplement: Supplementary file 1 — Table S1. Univariate Cox proportional hazards regression for overall and cancer-specific mortality of leiomyosarcoma and carcinosarcoma. Table S2. Multivariate Cox proportional hazards regression for non cancer-specific mortality of leiomyosarcoma or carcinosarcoma in 1:1 propensity score matching sample. Table S3. Multivariate Cox proportional hazards regression for non cancer-specific mortality of leiomyosarcoma or carcinosarcoma in 1:1 propensity score matching sample. (DOCX 22 kb) [file 12885_2019_5879_MOESM1_ESM.docx]

| **Additional file 1: Table S1. Univariate Cox proportional hazards regression for overall and cancer-specific mortality of leiomyosarcoma and carcinosarcoma** | | | | | | | | |
| --- | --- | --- | --- | --- | --- | --- | --- | --- |
|  | Leiomyosarcoma | | | | Carcinosarcoma | | | |
|  | Overall | | Cancer-specific | | Overall | | Cancer-specific | |
| Variables | HR (95% CI) | P-value | HR (95% CI) | P-value | HR (95% CI) | P-value | HR (95% CI) | P-value |
| Adjuvant radiotherapy |  |  |  |  |  |  |  |  |
| No treatment | reference |  |  |  | reference |  |  |  |
| EBRT alone | 0.92 (0.56, 1.50) | 0.740 | 0.88 (0.51, 1.55) | 0.666 | **0.63 (0.47, 0.85)** | **0.002** | 0.73 (0.51, 1.04) | 0.081 |
| Brachytherapy alone | 0.63 (0.16, 2.55) | 0.519 | 0.40 (0.06, 2.70) | 0.345 | **0.46 (0.32, 0.66)** | **<0.001** | **0.42 (0.25, 0.68)** | <0.001 |
| Combination radiotherapy ^a^ | **<0.01 (<0.01, <0.01)** | **<0.001** | **<0.01 (<0.01, <0.01)** | **<0.001** | **0.47 (0.29, 0.75)** | **0.001** | **0.53 (0.30, 0.95)** | **0.032** |
| Age |  |  |  |  |  |  |  |  |
| < 60 years | reference |  |  |  | reference |  |  |  |
| ≥ 60 years | **1.75 (1.26, 2.44)** | **<0.001** | 1.38 (0.94, 2.02) | 0.103 | **1.66 (1.24, 2.22)** | **<0.001** | 1.41 (0.99, 1.99) | 0.052 |
| Race |  |  |  |  |  |  |  |  |
| White | reference |  |  |  | reference |  |  |  |
| Black | **1.54 (1.04, 2.28)** | **0.032** | 1.48 (0.95, 2.31) | 0.082 | **1.43 (1.12, 1.81)** | **0.003** | **1.58 (1.17, 2.14)** | **0.003** |
| Others ^b^ | 0.98 (0.56, 1.72) | 0.953 | 0.84 (0.43, 1.64) | 0.614 | 1.00 (0.67, 1.49) | 0.996 | 0.95 (0.57, 1.59) | 0.857 |
| AJCC Stage |  |  |  |  |  |  |  |  |
| Stage I | reference |  |  |  | reference |  |  |  |
| Stage II | **2.04 (1.32, 3.14)** | **0.001** | **1.94 (1.17, 3.23)** | **0.011** | 1.04 (0.66, 1.64) | 0.857 | 1.08 (0.61, 1.93) | 0.784 |
| Stage III | **3.53 (2.35, 5.31)** | **<0.001** | **3.63 (2.30, 5.73)** | **<0.001** | **2.56 (2.06, 3.18)** | **<0.001** | **2.91 (2.20, 3.83)** | **<0.001** |
| Grade |  |  |  |  |  |  |  |  |
| Grade 1 | reference |  |  |  | reference |  |  |  |
| Grade 2 | 2.04 (0.23, 18.06) | 0.521 | 1.22 (0.13, 11.99) | 0.863 | 0.34 (0.07, 1.73) | 0.192 | 0.26 (0.05, 1.42) | 0.119 |
| Grade 3 | **8.89 (1.16, 68.08)** | **0.036** | 7.22 (0.93, 55.97) | 0.059 | 1.10 (0.28, 4.33) | 0.895 | 0.71 (0.18, 2.79) | 0.619 |
| Undifferentiated/anaplastic | 6.65 (0.88, 50.25) | 0.066 | 5.48 (0.72, 41.72) | 0.101 | 1.08 (0.27, 4.28) | 0.918 | 0.74 (0.19, 2.97) | 0.674 |
| Tumor size |  |  |  |  |  |  |  |  |
| ≤ 50 mm | reference |  |  |  | reference |  |  |  |
| > 50 mm | **1.74 (1.03, 2.95)** | **0.039** | **3.33 (1.55, 7.17)** | **0.002** | **2.23 (1.72, 2.90)** | **<0.001** | **2.45 (1.75, 3.43)** | **<0.001** |
| Lymphadenectomy |  |  |  |  |  |  |  |  |
| No | reference |  |  |  | reference |  |  |  |
| Yes | 1.14 (0.82, 1.60) | 0.435 | 1.14 (0.78, 1.67) | 0.495 | **0.53 (0.41, 0.68)** | **<0.001** | **0.58 (0.42, 0.79)** | **<0.001** |
| aHR, adjusted hazard ratio; AJCC, American Joint Committee on Cancer; CI, confidence interval; EBRT, external beam radiation therapy. | | | | | | | | |
| ^a^ Combination radiotherapy is EBRT with radioactive implants. | | | | | | | | |
| ^b^ Including American Indian, Alaska native, Asian Pacific Islander, and other unspecified. | | | | | | | | |
| Unknown data for adjuvant radiotherapy, race, AJCC stage, grade, tumor size, and lymphadenectomy status were not shown in the table. | | | | | | | | |

| **Additional file 1: Table S2. Multivariate Cox proportional hazards regression for non cancer-specific mortality of leiomyosarcoma or carcinosarcoma in 1:1 propensity score matching sample** | | | | | |
| --- | --- | --- | --- | --- | --- |
| Variables | Leiomyosarcoma (n = 180) | | Carcinosarcoma (n = 856) | | |
|  | Non cancer-specific ^b^ | | Non cancer-specific ^b^ | | |
|  | aHR (95% CI) | P-value | aHR (95% CI) | P-value |  |
| Adjuvant radiotherapy |  |  |  |  |  |
| No treatment | reference |  | reference |  |  |
| EBRT alone | 1.21 (0.14, 10.72) | 0.862 | **0.49 (0.26, 0.92)** | **0.027** |  |
| Brachytherapy alone | 4.48 (0.18, 108.96) | 0.997 | 0.63 (0.32, 1.25) | 0.182 |  |
| Combination radiotherapy ^a^ | NA |  | **0.24 (0.07, 0.79)** | **0.019** |  |
| aHR, adjusted hazard ratio; CI, confidence interval; EBRT, external beam radiation therapy. | | | | | |
| ^a^ Combination radiotherapy is EBRT with radioactive implants. | | | | | |
| ^b^ Model was adjusted by race. | | | | | |
| NA, no available because no patient died during the study period. | | | | | |
| Unknown data for adjuvant radiotherapy was not shown in the table. | | | | | |

| **Additional file 1: Table S3. Multivariate Cox proportional hazards regression for non cancer-specific mortality of leiomyosarcoma or carcinosarcoma in 1:1 propensity score matching sample** | | | | | |
| --- | --- | --- | --- | --- | --- |
| Variables | Leiomyosarcoma (n = 180) | | Carcinosarcoma (n = 856) | | |
|  | Non cancer-specific ^a^ | | Non cancer-specific ^a^ | | |
|  | aHR (95% CI) | P-value | aHR (95% CI) | P-value |  |
| Adjuvant radiotherapy |  |  |  |  |  |
| No treatment | reference |  | reference |  |  |
| Received Adjuvant radiotherapy | 1.27 (0.17, 9.85) | 0.817 | **0.48 (0.30, 0.78)** | **0.003** |  |
| aHR, adjusted hazard ratio; CI, confidence interval. | | | | | |
| ^a^ Model was adjusted by race. | | | | | |
| Unknown data for adjuvant radiotherapy was not shown in the table. | | | | | |
